# Urinary biomarkers for amyotrophic lateral sclerosis: candidates, opportunities and considerations

**DOI:** 10.1093/braincomms/fcad287

**Published:** 2023-10-24

**Authors:** Mary-Louise Rogers, David W Schultz, Vassilios Karnaros, Stephanie R Shepheard

**Affiliations:** Flinders Health and Medical Research Institute, College of Medicine and Public Health, Flinders University, Adelaide 5042, South Australia, Australia; Neurology Department and MND Clinic, Flinders Medical Centre, Adelaide 5042, South Australia, Australia; Flinders Health and Medical Research Institute, College of Medicine and Public Health, Flinders University, Adelaide 5042, South Australia, Australia; Flinders Health and Medical Research Institute, College of Medicine and Public Health, Flinders University, Adelaide 5042, South Australia, Australia

**Keywords:** ALS, urine, biomarker, proteins, metabolites

## Abstract

Amyotrophic lateral sclerosis is a relentless neurodegenerative disease that is mostly fatal within 3–5 years and is diagnosed on evidence of progressive upper and lower motor neuron degeneration. Around 15% of those with amyotrophic lateral sclerosis also have frontotemporal degeneration, and gene mutations account for ∼10%. Amyotrophic lateral sclerosis is a variable heterogeneous disease, and it is becoming increasingly clear that numerous different disease processes culminate in the final degeneration of motor neurons. There is a profound need to clearly articulate and measure pathological process that occurs. Such information is needed to tailor treatments to individuals with amyotrophic lateral sclerosis according to an individual’s pathological fingerprint. For new candidate therapies, there is also a need for methods to select patients according to expected treatment outcomes and measure the success, or not, of treatments. Biomarkers are essential tools to fulfil these needs, and urine is a rich source for candidate biofluid biomarkers. This review will describe promising candidate urinary biomarkers of amyotrophic lateral sclerosis and other possible urinary candidates in future areas of investigation as well as the limitations of urinary biomarkers.

## Teaser

Finding objective fluid-based biomarkers for amyotrophic lateral sclerosis that reflect the pathological processes occurring in a phenotypically heterogeneous disease is essential for assessing if treatments are working or not. This requires evaluating alternate non-invasive biofluids such as urine, with caveats such as standardizing collection, processing and validating candidates.

## Graphical Abstract

**Graphical Abstract:**
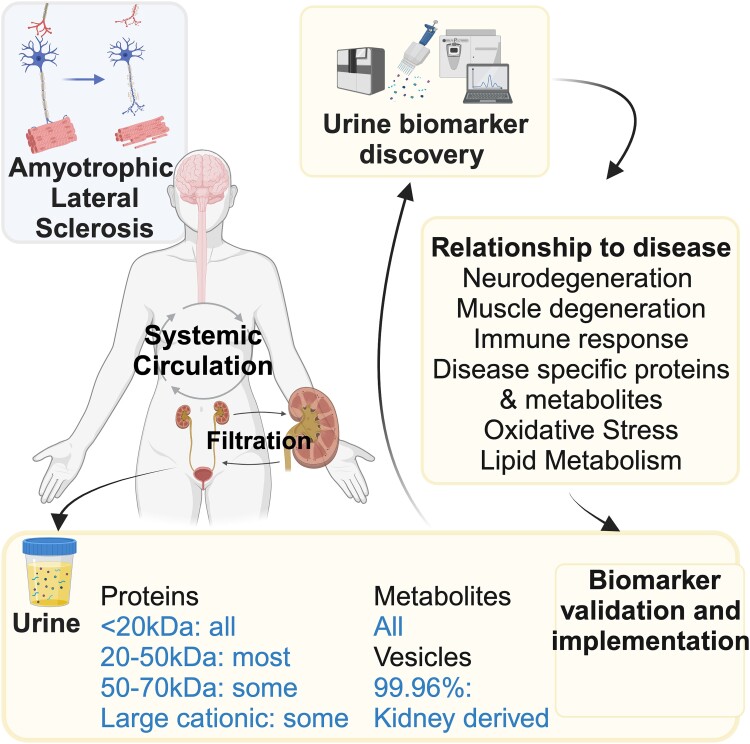

## Introduction

Amyotrophic lateral sclerosis (ALS) is a relentlessly progressive disease resulting in the death of the upper and lower motor neurons, around 15% of those with ALS also have frontotemporal degeneration (ALS/FTD), and gene mutations account for ∼10% of ALS.^1^ Numerous different disease processes culminate in the final degeneration of upper and lower motor neurons that can be fatal within 2–5 years after diagnosis. Some people diagnosed with ALS survive longer than 4–5 years, and others experience a much more rapid disease progression.^1,2^ This phenotype variability reflects a highly complex disease, where tools to group people at diagnosis, those that respond to specific treatments and those that can predict outcomes in clinical trials are crucial. Such tools are biomarkers, and numerous types are needed to report on the intertwined pathological processes occurring in a particular patient with ALS and develop personalized treatments.^2^ The National Institute of Health originally defined biomarkers as ‘measurable characteristics, indicative of biological, pathological or pharmacodynamic responses to therapeutic interventions’.^3^ The Food and Drug Administration in conjunction with the National Institute of Health has since produced a guidance document that summarizes each type of biomarker tool (diagnostic, monitoring, response or pharmacodynamic, predictive and prognostic), and the ways to ensure the tool are adequate for its proposed purpose, i.e. validation.^4^ These tools can be physical, physiological, imaging, genetic or biofluid tests.

Although numerous candidate prognostic, predictive and pharmacodynamic biomarkers for use in ALS have been identified, only the revised ALS functional rating scale (ALSFRS-R) has progressed to use in clinical trials for ALS.^5,6^ The ALSFRS-R is a questionnaire-based scoring scale in which 12 activities of daily living are scored from 0 to 4 (4 is normal) and summed to produce a score between 48 (i.e. healthy) and 0.^7,8^ Candidate biomarkers include electrophysiological, neurophysiological, tissue sampling, imaging, genetic and biofluid biomarkers. Those present in biofluids show great potential as a source for objective candidates in ALS. They are generally advantageous over electrophysiological, neurophysiological, tissue analysis and imaging markers in that testing is often easier for the participant, may incur lower expense and allows for reproducible quantification.^2,9-11^ Biofluid candidates are being increasingly tested alongside candidate treatments in adaptive platform trials, such as the HEALEY ALS Platform trial for ALS,^12^ with the same protocol and a shared placebo group used across multiple treatments being tested.

## Why urine? Components, comparison of urine to CSF and blood

The source of biofluid for ALS biomarker investigation is an important consideration. CSF is close to motor neuron injury, and neuroinflammation in ALS and for those reasons has been thought to have advantages as an ALS biomarker source, over systemic biomarkers such as blood and urine. However, if biomarkers found in urine and blood can be shown to be related to motor neuron degeneration and neurological status, they also should be investigated. This is shown in biomarkers such as neurofilament light (NfL)^13-15^ and interleukin (IL)-18^16^ being as useful in blood as they are as CSF biomarkers. Urine with a less complex proteome than blood and retaining the metabolome is an attractive and underinvestigated source of systemic biomarkers for ALS. Other advantages of urine are that large quantities can be collected, and it is less invasive for participants, when compared with CSF, especially for repeated collection.

**Figure 1:**
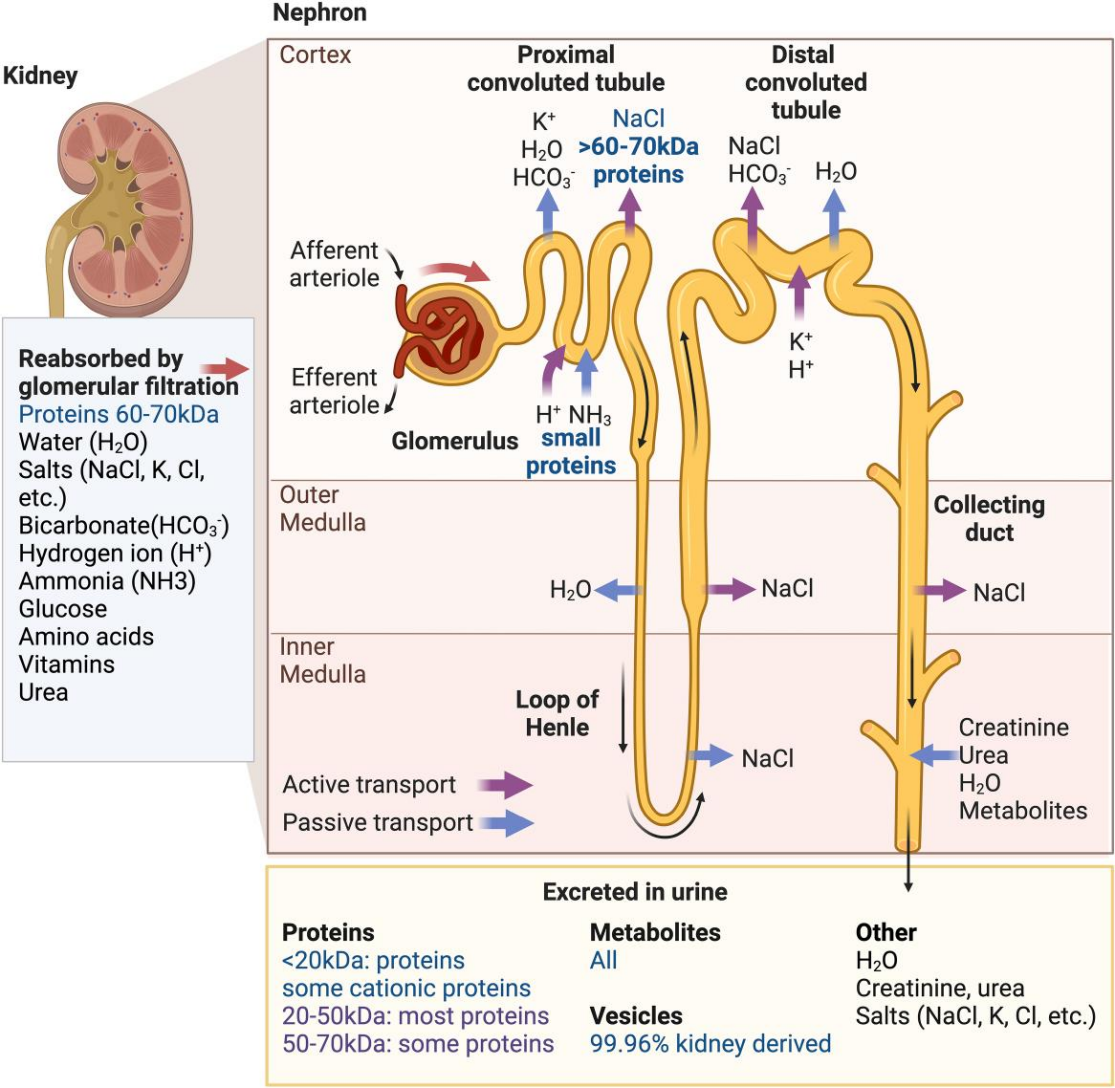
**Urinary biomarkers for ALS derived from normal kidney function**. Urine is a rich source of candidate ALS biomarkers, whose solute content is determined by the size and charge of components passing through the nephrons. The functional unit of the kidney is the nephron that consists of the glomerulus, which filters blood, and the renal tubule, which reabsorbs necessary components back into the blood. These components include salts like sodium chloride (NaCl), potassium (K^+^) and chloride (Cl^−^) ions. Hydrogen (H^+^), ammonia (NH_3_^+^) and bicarbonate (HCO_3_^−^) are among other ions that are reabsorbed, alongside glucose, amino acids, vitamins and urea. All the blood metabolites and proteins less than 20 kDa in size pass through the nephron and into the urine. Most proteins of 20–50 kDa size are excreted, while only some 50–70 kDa proteins, especially cationic proteins, are excreted. Most proteins larger than 60–70 kDa are actively transported back into blood at the proximal convoluted tubule. Levels of other urinary components such as salts and urea are determined by their active and passive transport along the tubule, while most vesicles found in urine are derived from the urogenital system.

Urine consists of components from the circulation, which are filtered through the kidney into the bladder and then excreted via the urethra. The human kidney (Fig. 1) is composed of more than 1 million functional units called nephrons, which are subdivided into two parts: the glomerulus, filtering plasma from renal blood flow, and the renal tubule, playing a vital role in the reabsorption of nutrients, fluids and other compounds that the body needs back to the blood. Approximately 180 L of fluid a day is filtered from renal blood flow that allows for toxins, metabolic waste products and excess ions and electrolytes to be excreted while keeping essential substances in the blood.^17,18^ Clearance of solutes occurs through a combination of glomerular filtration, tubular secretion and tubular reabsorption while maintaining plasma homeostasis.^17,19^ Urine is collected in the bladder and voided via the urethra. Human urine comprises water (95%), urea (2%), creatinine (0.1%), uric acid (0.03%) and lower levels of chloride, sodium, potassium, sulphate, ammonium, phosphate, other ions and molecules (including metabolites and protein) and cells and extracellular/microvesicles.^20-23^ Physiologically, 20–30% of proteins present in the blood appear in urine, whereas metabolites are freely filtered into the urine in exact proportion to that present in blood^24^ (Fig. 1).

## Urinary proteome

Although human urine is easy to obtain non-invasively, proteomic analysis and biomarker discovery have lagged that of serum/plasma, which has much more complex proteome and contains approximately 60–70 mg protein/ml.^25^ Over 99% of the blood proteome is composed of 22 high-abundance proteins (e.g. albumin, transferrin and immunoglobulins) unrelated to most disease states, making it difficult to identify low-abundance biomarkers by conventional mass spectrometry and enzyme-linked immunosorbent assay (ELISA).^26^ These high-abundance proteins are also found in the CSF.^27^ Conversely, human urine rarely exceeds 0.2 mg protein/ml^17,18^ because the renal system efficiently reabsorbs most proteins. In the final voided urine, protein does not exceed ∼150 mg/day, of which albumin accounts for 20 mg^17^ meaning it is often easier to identify low-abundance proteins.

An important consideration in surveying urine for biomarkers is our knowledge about how proteins enter the final urinary matrix (Fig. 1). Our understanding dating back to the 1960s indicates that the kidney’s filtration system progressively restricts the size of molecules excreted in the urine.^28^ All proteins with a molecular weight of 20 kDa or less easily cross the filtration barrier and those with a molecular weight of 20–50 kDa are mostly filtered into the urine. As the molecular mass of a protein increases, the fraction that is filtered progressively decreases such that compounds of 60–70 kDa are largely retained in the capillary lumen and reabsorbed. More recently, it has been shown that the charge of the proteins also impacts the size of the proteins excreted. Positively charged proteins (cationic) are more freely filtered than negatively charged (anionic) proteins as the glomerular barrier is anionically charged by glycosaminoglycan heparan sulphate in the glomerular basement membrane.^29^ Thus, anionic molecules of the same molecular weight are filtered to only 50% of those with cationic charge. Hence, the cut-off of 50 kDa may be larger if the protein is positively charged.^30^ Around 20–30% of proteins found in blood are also found in urine. There are also soluble proteins secreted by epithelial cells and extracellular vesicles (EVs) that all arise from the urogenital system and are excreted in urine.^31^ Despite the restriction on protein species that comes from the circulation, urine is a rich source of proteins/peptides for biomarker discovery. A caveat is the reported heterogeneity according to time of day, sex, age, diet, pH (4–8), proteolysis while the urine is stored in the bladder and degradation of collected urine samples upon storage.^32-34^ In a quantitative analysis of variability in the normal urinary proteome, inter-individual variability exceeded 47% and intra-individual variability exceeded 45%.^32^ Standardized protocols are essential to reduce variability in the urinary proteome, the most important being the time of collection and the time between collection and sample processing.^32^

As of 2023, there have been 4500 potential urinary protein groups identified and confirmed (UniProt search); many were found because of recent improvements in mass spectrometry detection and instrumentation. Practically, around 2000 protein groups in urine can be found using mass spectrometry.^35,36^ This can be contrasted with blood that contains an estimated possible 4500 protein groups,^37^ but only 300–600 are usually detectable, with a similar profile in CSF (proteinatlas.org/humanproteome). The number of groups of proteins identified in urine is clearly higher than in blood or CSF.^32-34^ This is mainly due to urine having a less complex proteome; it is not dominated by proteins with high abundance as is the case for blood^26^ or CSF^27^ but instead contains a wide range of low-abundance proteins.^34^ This source of biomarkers for ALS is relatively unexplored. Table 1 lists current ALS protein biomarkers, including those found in urine, such as size, relationship to pathology and neurological status and if upregulated or downregulated in ALS.

**Table 1:** Protein biomarkers of ALS in biofluids

Name, accession number	Pathological association	Size (kDa)	Biofluid	Change in ALS	Detection technique	Link to disease
p75^ECD^, P08138	Neurodegeneration	30–50	Urine	Increase	WB, LC-MS, ELISA	Prognostic, correlated to ALSFRS-R, increases longitudinally over disease progression.^38-40^
NfL, P07196	Neurodegeneration	61–68, 22	CSF, serum, blood	Increase	Immunoassay, ELISA, SIMOA, WB	Prognostic, correlated to ALSFRS-R and progression rate.^15,41-44^ NA for whole blood WB.^45^
pNfH, P12036	Neurodegeneration	190–210	CSF, serum	Increase	ELISA, SIMOA	Prognostic, correlated to ALSFRS-R and progression rate.^15,41,46,47^
Titin, Q8WZ42	Muscle degeneration	∼23	Urine	Increase	ELISA	Prognostic, especially for lower limb ALS.^48^
C9ORF72: poly-glycine-proline, NA	Disease specific	ND	CSF	Increase	ELISA	Related to repeats,^49-51^ not disease.^50^
TDP-43, ratio pTDP: TDP, Q13148	Disease specific	45, 43–50	Plasma, CSF	Increase or NA	SIMOA, WB, LC- MS, ELISA	Improves diagnosis in combination with NfL in SIMOA assay.^52^ Relationship to disease NA.^53,54^ Ratio pTDP: TDP, no correlation to disease.^55^
FUS, P35637	Disease specific	53	Plasma	Increase	WB	Relationship to disease not investigated.^54^
SOD1, P00441	Disease specific	15.4	Plasma	Increase	WB	Relationship to disease not investigated.^54^
pTau and tTau, P10636	Other	40–60	CSF	Increase	Immunoassay	tTau and pTau/tTau prognostic.^56^
Collagen, P53420	Other	164	Serum, urine	Decrease	Radioimmunoassay	Correlated to disease duration.^57,58^
MCP-1, P13500	Immune system	11–13	CSF, serum	Increase, NS	ELISA	CSF prognostic.^16,59-61^
CXCL13, O43927	Immune system	13	CSF	Decrease	Immunoassay	Relationship to prognosis and ALSFRS-R not investigated.^62^
CHIT1, Q13231	Immune system	51	CSF	Increase	LC-MS, ELISA	Prognostic, correlated to disease progression.^59,63-66^
CHI3L1, P36222	Immune system	40	CSF	Increase	LC-MS, ELISA	Prognostic, correlated to progression.^59,65,66^
CHI3L2, Q15782	Immune system	39	CSF	Increase	LC-MS	Correlates to disease progression.^66^
C reactive protein, P02741	Immune system	22	CSF, serum	Increase/NS	LC-M, ELISA/mesoscale	Significant in CSF.^67^ NS in CSF/serum.^16^ Serum levels increased in ALS;^68^ prognostic.^69^
IL-6, P05231	Immune system	21–28	CSF, serum	Increase/NS	ELISA/immunoassay	CSF correlated to duration^70^/NS.^16^ Increased in serum.^71^
IL-18, Q14116	Immune system	23	CSF	Increase	Immunoassay	Correlated to progression and changes over progression.^16^
TNF-α, P01375	Immune	17	CSF, serum	Increase	Immunoassay	Not consistently associated with ALS prognosis or progression.^71,72^
Cystatin C, P01034	Immune system	13.3	CSF	Decrease	ELISA	Correlated to survival, prognostic.^73-75^
Native transthyretin, CysGly, P02766	Immune system	55 69	CSF CSF	Decrease Increase	LC-MS	Relationship to ALS not tested.^76^ CysGly—transthyretin does not change over ALS progression.^67^
sCD14, P08571	Immune system	40	CSF, serum, urine	Decrease Increase	ELISA	Lower CSF CD14 associated with poor prognosis.^77^ Elevated in CSF, urine and serum. Serum sCD14 prognostic.^68^

LC, liquid chromatography; MS, mass spectrometry; NA, not applicable; NS, not significant; WB, western blot.

## Urinary protein candidates that have been identified as ALS biomarkers or candidates in other biofluids

### Related to neurodegeneration

#### p75^ECD^

A neurodegeneration biomarker identified in urine is the extracellular domain (ECD) of the common neurotrophin receptor p75 (p75^ECD^). The full-length receptor, p75NTR, is the 16th member of the tumour necrosis factor (TNF) superfamily. It is a 75 kDa transmembrane protein that binds immature (pro) or mature neurotrophins (brain-derived growth factor, nerve growth factor, neurotrophin 3 and neurotrophin 4/5). This receptor is involved in promoting cell survival or apoptosis dependent on its ligands, how it complexes with other cell receptors (e.g. tyrosine kinase receptors A, B and C and sortilin), cell type and surrounding environment.^78,79^ The result is that p75NTR is involved in processes such as neuron development, pruning and maintenance of nerve cells. Importantly, for ALS, p75NTR is expressed by motor neurons during development, only to be downregulated in adulthood and only re-expressed by motor neurons and Schwann cells following neuronal damage such as that in ALS animal models^80,81^ and in people with ALS.^80,82,83^ Additionally, p75NTR undergoes regulated intramembrane proteolysis, in which the 50 kDa ECD of the 75 kDa receptor is cleaved by TNF-α-converting enzyme, α-secretase/ADAM10/17.^79,84^ In the 1980s, DiStefano *et al*.^85^ described the appearance of the ECD in rat urine via radioimmunoassay, post-bilateral sciatic nerve lesions. This observation was followed up in our laboratory using immunoprecipitation experiments on SOD1G93A mouse urine to show the appearance of ECD in an ALS animal model.^38^ Further immunoprecipitation experiments showed that humans with ALS also have ECD fragments in their urine at a molecular weight of 35–50 kDa, with the presence of ECD confirmed by mass spectrometry.^38^ An ELISA was then developed to measure mouse and human urinary p75^ECD^, and this assay showed increased levels of ECD in people with ALS compared with healthy controls.^38^ Using this ELISA, p75^ECD^ was further found to be a candidate biomarker of disease progression in ALS and baseline p75^ECD^ was prognostic for survival and correlated to disease severity described using the ALSFRS-R.^39,40^ Hence, urinary p75^ECD^ can be classified as a monitoring and prognostic biomarker.

Recently, urinary p75^ECD^ testing has been included as an exploratory biomarker in several clinical trials.^86,87^ Notably, in a Phase 2a safety trial of the antiretroviral Triumeq, although not designed to determine efficacy, urinary p75^ECD^ levels over the treatment period showed potential as a biomarker.^86^ A follow-up randomized clinical trial is underway where urinary p75^ECD^ has been included as a potential biomarker of efficacy (NCT05193994).

#### Neurofilament light and heavy chains, not yet identified in urine

Neurofilaments are important structural components of myelinated axons and help to increase axon diameter, allowing for faster nerve conductance. In the CNS, neurofilaments are protein polymers composed of NfL (61–68 kDa) and alpha-internexin (68–72 kDa), which form the neurofilament core and co-assemble with neurofilament medium chain (∼150 kDa) and phosphorylated neurofilament heavy chain (pNfH, 190–210 kDa).^41,46,47^ All four proteins contain conserved rod domains and unique amino-terminal (N-terminal) and carboxyl-terminal (C-terminal) domains.^46^ Of these proteins, NfL is a well-established marker in CSF and serum of both acute and chronic neuronal damage, is increased in ALS, frontotemporal degeneration (FTD) and a number of diseases involving neuronal damage^13,42-44,88-90^ and is recognized as a neurodegenerative biomarker for ALS.^15,91,92^ pNfH in CSF and serum has also been demonstrated as a neurodegenerative biomarker for ALS.^15,41,46,47^ The specificity and sensitivity of the single molecule array (SIMOA) in comparison with ELISA have allowed accurate measurement of NfL^14,15,93,94^ and recently pNfH.^15^ Furthermore, NfL has been validated as a biomarker for clinical trials of familial ALS.^92,95^

Although detectable by ELISA or SIMOA, interestingly, NfL has not been readily detected by conventional shotgun mass spectrometry methods in the CSF^63^ or blood^96,97^ of ALS patients. One explanation is that NfL is at very low concentrations in CSF (100–500 pg/ml range).^44,98,99^ The levels in peripheral blood (10–100 pg/ml range) are even lower^13,15^ and may be masked by the high-abundance proteins in serum that make up 99% of the proteome.^26,100^ This is evidenced by the detection of NfL by mass spectrometry as significantly upregulated in the blood of ALS patients after depleting high-abundance proteins and enriching tissue-derived proteins^101^ and upregulated in CSF after using isobaric tags.^102^

Another explanation as to why it is difficult to identify NfL in mass spectrometry of blood and CSF is that the stability and fragment state of NfL has not been considered and may influence detection. In a normal state, it has been widely recognized that full-length NfL is slow to turn over. This was demonstrated in rodents^103^ and in retinal ganglion cells.^104^ NfL is, however, degraded by the calcium protease calpain,^105,106^ and degradation is influenced by phosphorylation state.^106^ Protease activity and phosphorylation are important in axonal degradation, but there is no indication as to the proportion of full-length and other NfL species in CSF and blood in ALS. There may be multiple NfL species as axons degrade as suggested by a recent study using immunocapture coupled with mass spectrometry where multiple molecular weight species of NfL were identified in Alzheimer’s CSF, but no full-length NfL was detected.^107^ Intriguingly, Malaspina *et al*.^45^ also found the main NfL band in plasma samples from ALS patients on a western blot was 22 kDa (not 61 kDa). It could be speculated that accurate serum NfL measurement from ALS patients by SIMOA means that it is not necessary to consider half-life and degradation. However, if the level of phosphorylation and protease activity is driving NfL accumulation, it is important to determine the most important NfL fragments that relate to ALS. The immunocapture study in CSF from Alzheimer’s patients indicated that the antibody used in the commercial NfL SIMOA targets a small peptide of eight amino acids: NFL 324–331. It would be interesting to determine the truncated species present in ALS CSF and blood. It would be unlikely that the 61 kDa NfL could enter the urine, as its size means it would be restricted and most likely reabsorbed.^28-30^ However, fragments of NfL may be present in urine and require further investigation. Interestingly, the only known study where urinary NfL was measured by SIMOA found no differences between urinary NfL in 93 people with stroke/haemorrhage disease compared with 10 healthy controls when corrected for urinary dilution.^108^ The form of NfL fragments in urine should be investigated and compared with blood and CSF, to determine if fragments of NfL are important as a urinary ALS biomarker.

### Related to muscle degeneration

#### N-terminal titin

Another promising urinary candidate protein is the N-terminal fragment of titin, which is a marker of muscle degeneration. Titin (originally known as connectin) is a major myofibrillar component of skeletal muscle.^109^ Titin is large (3200–4200 kDa) and responsible for passive muscle elasticity. N- and C-terminal fragments of titin are produced through cleavage by matrix metalloproteinases-9 or -2 and calpain and have been detected in urine by proteomic studies.^110,111^ Matrix metalloproteinases are associated with the degradation of cardiac titin and calpain with skeletal muscle titin. Interestingly, a wide range of titin peptides covering the whole molecule are found in the urine of Duchenne muscular dystrophy model mice.^112^ Human and mouse ELISAs have been developed for the N-terminal fragment (∼23 kDa)^113,114^ and have revealed that the N-terminal fragment is increased in cardiac patients,^115^ pathological conditions such as oxidative stress and muscle loss including extensive literature in Duchenne muscular dystrophy.^116,117^ An interesting observation in Duchenne muscular dystrophy mouse studies was that there was an increase as muscular dystrophy peaks in early life and then a sharp decrease with age and muscle regeneration, with either creatinine or specific gravity as urinary dilution correction factors.^114,118^

Urinary N-terminal titin was suggested as a novel biomarker for ALS with strong survival and prognostic potential.^48^ It was also suggested as a progression biomarker, but, unlike urinary p75^ECD^, where sampling was every 3 months across disease progression,^39,40^ there was only one follow-up sample at 6 months,^48^ and no rate of progression reported, so its role as a progression biomarker is not clear. An earlier study looking at fragments of titin via western blot showed that people with ALS, Charcot–Marie–Tooth disease, limb girdle muscular dystrophy and myotonic dystrophy patients had minimal reactive fragments when compared with Duchenne muscular dystrophy.^119^ A recent publication has demonstrated in an animal model that urinary N-terminal fragments of titin (ratio to creatinine) do not increase post-sciatic nerve injury and urinary measures may not reflect early muscle denervation events.^120^ This suggests that urinary N-terminal titin may not be a marker of early or minor muscle degeneration and further investigation is required into the muscle loss required before urinary titin is detected in ALS. The literature so far suggests that urinary N-terminal fragments of titin are possible prognostic biomarkers for ALS. However, further investigation into titin over ALS progression and a comparison of urinary correction factors (i.e. specific gravity, osmolality and creatinine) is required. This is because muscle loss may involve reduced urinary creatinine when measured over 24 h, but this is not so clear in spot urine samples.^121-123^

### Extracellular vesicles

EVs are nanosized, are released from cells, consist of a lipid membrane and contain cargo including DNA, RNA, proteins, lipids, amino acids and metabolites (microvesicles.org). Upon their discovery in biofluids,^124^ EVs were thought to remove intracellular waste, but later studies found they contain mRNA and miRNA and recipient cells can translate EV-associated mRNAs into proteins.^125-127^ Larger EVs called microvesicles (also known as ectosomes, microparticles, oncosomes or shedding vesicles) have a diameter of ∼100–1000 nm and are shed by budding of the plasma membrane; smaller EVs or exosomes are ∼30–140 nm in size, originate in the endosome and are released through exocytosis.^128,129^ Apoptotic bodies (∼800–5000 nm) are released during cell death and contain nuclear material, cellular organelles and membrane and cytosolic contents^128,129^

EVs and their contents make interesting candidate biomarker sources. EV contents remain stable due to the phospholipid bilayer membrane, and EVs have been isolated from CSF,^130^ blood^131^ and urine.^23^ It is important to consider that urine EVs are mostly derived from the kidney, bladder and genital tissues. Collecting duct epithelial cells secrete exosomes, but there is no evidence that endogenous non-urinary-derived EVs reach the urine under physiological conditions.^132^ In a large study, it was found that only 0.04% of urinary EV (uEV) proteins were derived from outside the urinary tract in healthy individuals.^132^ Nevertheless, global changes in the contents of urinary exosomes because of genetic disease have been proposed. For example, an increase in urinary exosome-associated phosphorylated Ser-1292 leucine-rich repeat kinase 2 (LRRK2; 286 kDa) may be a biomarker for familial Parkinson’s disease, and an increase in urinary pS1292-LRRK2 may be associated with a higher risk of converting to Parkinson’s.^133^ Disease-specific proteins of ALS (as mentioned below) would not be expected to be in uEVs unless associated with familial ALS and expressed in epithelial cells. Other miRNA, DNA, lipids and metabolites are also found in uEVs but, unless there is a body system-wide change, would not be likely to be biomarkers of ALS.

### Disease-specific proteins

#### TDP-43

Ubiquitinated hyperphosphorylated cytoplasmic inclusions of the 43 kDa transactive response DNA-binding protein (TDP-43) is the pathological hallmark of ALS in 96% of all ALS cases and 50% of cases of FTD. TDP-43 is present in cytoplasmic inclusions and is not normally excreted,^134^ and its measurement in CSF and blood has shown a limited value as a diagnostic and prognostic biomarker, showing high variability in the results of different studies using ELISA,^135-137^ but clearly elevated using SIMOA as a more sensitive assay.^52^ Clearance of pathological inclusions may include an accumulation of both phosphorylated and total TDP-43 in the CSF, and measurement by ELISA/SIMOA specific for phosphorylated TDP-43 in a ratio with total TDP-43 may be more reliable as a biomarker in plasma.^55^ A mass spectrometry method developed by Turner *et al*. indicated intracellular brain-derived TDP-43 as a possible ALS biomarker based on the ratio of C:N terminal peptide fragment detection.^138^ Since it is not normally excreted, an attractive explanation is that pathological TDP-43 is sequestered from cells via microvesicles or EVs.^53,54,139^ It may be the EVs that enable a prion-like propagation of TDP-43 inclusions as alluded to by Don Cleveland’s group.^134^ TDP-43 has not been documented in urine, but the full-length 43 kDa and the 35 and 25 kDa C-terminal fragments may be at very low concentrations, which requires enrichment strategies for mass spectrometry detection. Since there has been no definitive proof uEV (proteins derived from outside the urinary system with the vast majority from epithelial cells of the collecting duct),^132^ it would be unlikely that pathological TDP43 inclusions that derive from the CNS would be in uEVs. Nevertheless, uEvs could contain mutant TDP43, but since this protein is not highly expressed in adult tissue,^140,141^ it may be difficult to detect.

#### C9ORF72 dipeptide repeat proteins

Pathologically, *C9orf72* is the most common gene implicated in ALS and FTD affecting 40% of familial ALS and 25% of familial behavioural-variant FTD. A pathological mechanism of *C9orf72* gene expansion entails the translation of the expansion into dipeptide repeat proteins (DPRs): glycine-alanine, glycine-arginine, proline-alanine, proline-arginine and glycine-proline (GP). Production of poly-proline-arginine, poly-G and poly-glycine-alanine leads to neurotoxicity via impaired protein translation.^49,142,143^ Dipeptide repeat proteins may be associated with phase separation^144^ that influences toxicity. Poly-glycine-proline in CSF, measured by ELISA, has attracted attention as a potential biomarker in C9orf72 gene expansion carriers in both behavioural-variant FTD and ALS.^49-51^ Asymptomatic mutation carriers have also been found to have elevated CSF poly-glycine-proline, and levels are raised in those diagnosed with ALS, in most, but not all mutation carriers.^50^ The size of the dipeptide repeat proteins is, however, unknown,^49^ nor if present in urine.

#### FUS/SOD1

ALS-causing mutations such as fused in sarcoma (FUS) and superoxide dismutase 1 (SOD1) conspicuously lack TDP-43 proteinopathy in most cases.^145^ Mutations are found in the FUS C-terminal nuclear localization sequence, causing the mislocalization of the normally nuclear protein to the cytoplasm, leading to the accumulation of cytoplasmic FUS and FUS aggregation.^146^ FUS is a 53 kDa protein, present in cellular inclusions, and has not been reported in body fluids including urine.

It was reported that EVs most likely contain mutant SOD1.^54^ There have been over 200 SOD1 mutations identified in ALS, and familial and sporadic SOD mutations account for about 5% of ALS. This type of familial ALS results in the death of motor neurons, similar to sporadic ALS, and is not caused by a change in enzyme activity.^2,147^ SOD1 is a 15.4 kDa protein and is ubiquitously found in human urine and difficult to distinguish between kidney derived and that from mutant SOD1 ALS-associated protein in ALS urine. uEVs that derive from kidney epithelium^132^ may contain mutant SOD1 or FUS but have not been investigated.

### Other proteins

#### Ratio of phosphorylated tau to tTau

Tau is a neuronal microtubule-associated protein that is present in several different isoforms (40–60 kDa) depending on post-translational modifications, including phosphorylation.^56^ Specific kinases mediate the phosphorylation of tau at threonine 181 (pTau), which lost its affinity for tubulin, leading to microtubule instability and disintegration. Total tau (tTau) and pTau have been proposed as biomarkers in neurodegenerative disorders. However, the clinical usefulness of tTau and pTau as diagnostic and prognostic biomarkers in ALS is still debated. There are various studies indicating that tau, ptau and tTau in CSF, is a candidate ALS biomarker^148^ or not.^149,150^ Studies have also shown CSF pTau/tTau ratio as an ALS biomarker,^56,148,151^ and this may be prognostic and diagnostic, differentiating patients with ALS from mimics.^56^ The type of tau in urine has not been investigated, but the size may restrict excretion.

#### Collagen type IV

Collagen type IV makes up about 50% of all basement membrane components^152,153^ and provides support to epithelium, endothelium, muscles, fat cells, Schwann cells and axons.^152^ Type IV collagen has three polypeptide α-chains in triple helix form (540 kDa), and because of its size, the only passage of entry into the urine is through leaky tubules in kidney injury and diabetes.^154^ In ALS, abnormalities in the basement membrane and its surrounding structures were reported in SOD1^G93A^ mice.^155^ In ALS patients, an immunohistochemical study found that there was decreased type IV collagen in the skin and in serum by radioimmunoassay.^57^ Interestingly, there was significantly less collagen IV in urine from a small study of 20 people with ALS compared with 20 healthy controls but no correction for creatinine.^58^ In ALS, a lower level of collagen IV compared with healthy controls contrasts with higher levels in kidney dysfunction and agrees with loss of collagen in the skin, and this is a systemic process. This is supported by not only a decrease in skin and urinary collagen but also less collagen metabolites such as the 486.5 Da glucosylgalactosyl hydroxylysine^156^ measured by reverse-phase high-performance liquid chromatography (HPLC) using a standard curve. Larger studies using ELISA and mass spectrometry HPLC should be undertaken to determine the diagnostic and prognostic ability of urinary collagen IV in ALS.

### Additional proteins related to ALS pathology

#### Progranulin, vascular endothelial growth factor, TGF-beta and ferritin/transferrin

Progranulin is a conserved 593 amino acid, 88 kDa glycosylated, secreted protein. Pathogenic mutations of the progranulin gene have been found associated with FTD (but not ALS) and result in reduced progranulin in the CSF.^157^ It is not changed in CSF of ALS compared with healthy controls,^158^ and there is no correlation between CSF and serum progranulin.^159^ Progranulin and granulin peptides produced by proteolysis that promotes inflammatory activity are produced by many types of tissue.^160^ Taken together, including the size of the whole molecule, the usefulness of urinary progranulin as a biomarker for ALS is not clear. Other growth factors involved in neurodegeneration such as vascular endothelial growth factor^72^ in CSF (21–27 kDa) and transforming growth factor-beta (44 kDa) and receptors in CSF and serum^100,161,162^ have been suggested as ALS biomarkers but have not been measured in urine. Blood-based ferritin and hepcidin associated with iron metabolism have also been proposed as biomarkers of ferroptosis in ALS, a process associated with oxidative stress.^163,164^ However, the kidney is actively involved in iron homeostasis as it reabsorbs filtered iron to prevent loss in the kidney such that it would be expected to be difficult to distinguish the relationship between serum and urine ferroptosis in people with normal kidney function and blood iron status.^165^

### Immune markers

The pathology of ALS results in damage to neurons, resulting in the innate and adaptive immune system responding to reduce the damage and then being overwhelmed. The innate system is triggered by aggregated proteins (e.g. TDP-43, FUS or SOD1) or danger signals (e.g. reactive oxygen species) produced by motor neurons. This results in reactive microglia/macrophages and astrocytes, the main components of the innate immune system in the CNS, responding in an anti-inflammatory manner, releasing, for example, neurotrophic factors. Intracellular nucleotide oligomerization domain-like receptor protein 3 inflammasome complexes in astrocytes also recognize and respond to misfolded proteins and mediate inflammatory responses. The adaptive immune system involving T cells is well described in ALS and Tregs, and M2 macrophages/microglia respond to danger signals by producing anti-inflammatory signals such as transforming growth factor-beta, IL-10, IGF-1 and IL-4, in the Th2 anti-inflammatory response and suppress T-helper type 1 cells in the case of Tregs.^166^ The adaptive response can thus be also observed in systemic change in ILs/chemokines. As the danger signals increase, the innate and adaptive anti-inflammatory response becomes overwhelmed, and there is a switch to a pro-inflammatory process, which results in the release of TNF-α, IL-6 and IL-1β and inflammasome-caspase 1, from glia and pro-inflammatory cytokines [e.g. ILs and interferon-gamma (IFN-γ)] from T-helper type 1 and 17 cells of the adaptive immune system that, in turn, worsens disease progression.^167^ Thus, there is an innate and adaptive response, and immune markers that reflect the stage of ALS if found in urine may be useful as prognostic, progression or predictive biomarkers for ALS.

#### MCP-1 and chemokines

A candidate biomarker for immune response in ALS is monocyte chemoattractant protein-1 (MCP-1) also known as chemokine C–C motif ligand 2. Chemokines are grouped into four classes based on the positioning of their N-terminal cysteine residues: CC, CXC, XC and CX3. MCP-1/chemokine C–C motif ligand 2 belongs to a sub-family of 27 CC chemokines with an N-terminal CC domain.^168^ It is a chemoattractant chemokine of 11–13 kDa that is involved in activating microglia and promoting the migration of peripheral immune cells such as monocytes/macrophages to inflammation sites.^169,170^ In the SOD1 mouse model of ALS, increased levels of MCP-1 are found in the spinal cord.^171^ Measurement of MCP-1 in CSF by ELISA has indicated that it is increased in ALS compared with disease mimics and healthy controls.^59^ However, levels are inconclusive in blood.^16,60,61^

Urinary MCP-1 is dysregulated in several kidney diseases and in diabetes.^172^ It has been suggested as a diagnostic biomarker for lupus nephritis^173^ and also for early kidney dysfunction and diabetic kidney disease.^174^ Urinary MCP-1 has also been suggested as a major indicator of pain^175^ and is increased in Alzheimer’s disease, but levels were also influenced by age and gender.^176^ Yet, there are no studies investigating MCP-1 in urine in ALS. A comparative study in CSF, blood and urine, in comparison with the ALSFRS-R, would determine the relevance of this marker for ALS prognosis and progression and as a predictive biomarker. Highlighting the need for other chemokines to be investigated is a recent report of chemokine CXCL-13 as a potential biomarker for ALS.^62^ Interestingly, the level of this marker declined in CSF and blood from ALS patients, sampled at various times from diagnosis. This interesting work could be repeated across biofluids at disease diagnosis, in comparison with the ALSFRS-R and over disease progression.

#### Chitinases

Chitin is a polysaccharide that is an essential structural component in numerous organisms. It is degraded by a chitinase, including chitotriosidase (CHIT1), an acidic mammalian chitinase, and several chitinase-like proteins: chitinase-3-like 1 (CHI3L1), chitinase-3-like 2 (CHI3L2), oviductin-specific glycoprotein and stabilin-1-interacting chitinase-like protein. CHIT1 (51 kDa), CHI3L1 (40 kDa) and CHI3L2 (39 kDa) have been investigated as biomarkers for ALS. CHIT1 and CHI3L2 are produced by macrophages, neutrophils and microglia, and CHI3L1 is also produced by reactive astrocytes.^64,65,177-179^ There has been quite extensive work examining these chemokines as prognostic biomarkers in CSF and blood but not urine.

CHIT1 was identified in CSF using mass spectrometry and found to be a candidate prognostic marker.^63,64^ A longitudinal mass spectrometry analysis indicated that CHIT1, CHI3L1 and CHI3L2 correlate with disease progression and with pNfH levels.^66^ Using commercially available ELISAs, Gille *et al*.^59^ showed that CHIT1 and CHI3L1 poorly discriminate between ALS and mimics and weakly correlate with disease progression. CSF CHI3L1 was independently associated with survival and suggested as a prognostic biomarker. However, it should also be noted that a CHIT1 polymorphism has been identified that reduces the CHIT1 levels in CSF of patients with ALS.^180^ A contrasting study using both mass spectrometry and ELISA showed that CSF CHIT1 was significantly higher in ALS compared with disease and healthy controls, while CHI3L1 was higher in ALS and disease controls than healthy controls, and the rate of increase in these biomarkers correlates to disease progression.^65^

Unlike CSF, there has been no significant increase in CHIT1 nor CHI3L1 in blood from ALS compared with controls nor association with disease.^60,64,65^ This suggests that CHIT1 and CHI3L1 may be site specific rather than systemic inflammatory markers. Neither CHIT1 nor CHI3L1 has been investigated in urine but may not be viable urinary candidates as neither is significantly elevated in the blood when compared with controls; this, along with the CHIT1 polymorphism, complicates the interpretation of results.

#### C-reactive protein

C-reactive protein (CRP) is an acute phase protein of 22 kDa produced in the liver and secreted into the bloodstream mostly during an inflammatory episode, largely in response to IL-6 (IL-6) signalling and, to a lesser extent, IL-1beta and other pro-inflammatory cytokines.^181^ CRP plays a variety of key roles during inflammation. It binds to damaged, necrotic and microbial cells, promotes phagocytosis by neutrophils and macrophages, and activates the complement system, which itself helps maintain inflammation. Rising CRP concentrations furthermore activate neutrophils and monocytes and promote the secretion of IL-6, IL-1β and TNF-α.^181^ Because of these effects, CRP has been classically regarded as a pro-inflammatory molecule. At the same time, CRP has anti-inflammatory effects: it stimulates the release of anti-inflammatory agents such as IL-10 and IL-1Rα, and, while activating the complement system, it also recruits several complement inhibitors, possibly in a time-dependent manner.^182^ As a result, the net effect of CRP in vivo appears to be weakly anti-inflammatory.^183^ In addition, IL-6 stimulating production of acute phase proteins such as CRP can occur in the absence of inflammation, rather than as in ALS as part of clearance of damaged cells.

There have been many apparent inconsistencies concerning the physiological roles of CRP including in ALS, which have been clarified by the discovery that this protein exists in two isoforms with different functions, a pentameric isoform synthesized by the liver (pCRP) that is largely anti-inflammatory and a monomeric isoform (mCRP) that is activated by local cues of inflammation and tissue injury and is pro-inflammatory.^183^ mCRP stimulates secretion of pro-inflammatory cytokines, induces the M1 phenotype in macrophages and promotes the release of reactive oxygen species, which function not only to debilitate pathogens but also to exact collateral damage on host tissue.^183,184^ While mCRP activates the complement, it also blocks the final stages of the cascade in the presence of certain inhibitory factors. This mechanism may permit a tightly controlled activation of the complement during the non-inflammatory removal of damaged cells.^182^

There have been a number of studies investigating CRP as a biomarker for ALS showing significant increases in CSF^67^ and serum^68,185,186^ or no significance in CSF and serum.^16^ A systematic review has since shown serum CRP is prognostic for ALS.^69^ CRP has been used to group patients in a Phase 2B clinical trial of an immune modulator NP001, where it was postulated that high CRP would mean a faster progression rate, but this criteria grouped those whom had a slower progression but were responsive to anti-inflammatory therapy.^187^ None of the literature looks at specifically looks at mCRP and pCRP, so it is difficult to distinguish pro-inflammatory or anti-inflammatory states. Since serum CRP is a routine general non-specific inflammatory marker, urinary CRP may not be useful.

#### ILs and other cytokines

ILs are one of the most well-reported indicators of systemic anti- and pro-inflammation. They are mainly synthesized by T cells, macrophages and endothelial cells, promoting the development and differentiation of T and B cells, and hematopoietic cells and range in size from 12–30 kDa. A number of these have been reported as changed in ALS, including IL-1β, IL-1Rα, IL-1, IL-2, IL-4, IL-5, IL-6, IL-7, IL-8, IL-9, IL-10, IL-12p70, IL-13, IL-15, IL-17, IL-17A, IL-18 and IL-21.^61,71^ It has not been clearly demonstrated that the serum/plasma levels of these markers increase (or decrease) at baseline in ALS.^64,65^ However, some ILs found in CSF may have some utility in the separation of ALS from disease mimics. IL-6 and IL-18 have been found upregulated in the CSF of ALS patients,^70^ and in a large progression study, IL-18 (but not IL-6) was consistently elevated across progression in the CSF of ALS patients.^16^ TNF-α is a 17 kDa major pro-inflammatory cytokine secreted by activated macrophages and is involved in the induction of cytokine production, phagocyte cell activation, activation or expression of adhesion molecules, and growth stimulation.^188^ TNF-α is reported upregulated in the CSF,^72^ and in serum,^71^ but not consistently associated with disease state. Increased urinary ILs IL6ST and IL-19 have been reported in a small study in Alzheimer’s,^189^ although the levels were not adjusted for dilution. However, there are no reports of urinary ILs as a biomarker in ALS. One reason may be that urinary IL levels are not a clear surrogate for blood levels. This is supported by a study showing no correlation between the levels of 13 cytokines in urine and plasma among a group of healthy, reproductive-aged women.^190^

#### Cst C and transthyretin

Cystatin C (Cst C) is a potent extracellular inhibitor of cysteine protease of ∼13.3 kDa and is generally considered a ubiquitously expressed protein^191^ used as a biomarker of kidney function. A decline in urine accompanies a rise in blood and failed kidney function. Independent of its inhibition of protease, it has also been implicated in apoptosis and inflammation.^192^ Cst C and transferrin are found in Bunina bodies, which are inclusion bodies found in lower motor neurons in ALS^193,194^ and may be present with but distinct from those with TDP-43 inclusions.^195^ Cst C levels are decreased in the CSF of ALS patients^73,74^ and correlated with the survival time implying that it is prognostic and may be a potent neuroprotective in ALS.^75^ The neuroprotective effect of Cst C has been shown in cell culture experiments where mutant SOD1 was expressed.^196^ However, Cst C level is a common signature of neuron vulnerabilities and neurodegeneration and is not specific for ALS.^197^ Urinary Cst C in ALS has not been investigated but would be difficult to distinguish from being a marker of kidney function.^192^ Transthyretin is a 55 kDa protein that is primarily synthesized by the liver and the choroid plexus.^198^ Reduced levels of native transthyretin^76^ and increased levels of oxidized CysGly–transthyretin are found in the CSF in ALS compared with controls^67^ and may be involved in dealing with TDP-43 inclusions.^199^ There are also several genetic mutations of transthyretin resulting in amyloid neuropathy that can mimic motor symptoms of ALS.^198^ Like Cst C, it is not specific for ALS. Urinary transthyretin has not been investigated, but the 55 kDa size means a large proportion may be largely retained in the capillary lumen of the kidney tubules and reabsorbed.^28^

#### Soluble CD14

Monocytes/microglia express CD14 early in ALS, preceding onset.^200^ There is also increased CD14 in spinal cord tissue in ALS patients.^171^ Soluble CD14 (sCD14) is a 40 kDa part of the CD14 receptor that is cleaved from the cell surface and released after monocyte activation. In a small study, CSF, blood and urinary sCD14 from ALS patients was found elevated when compared with healthy controls and blood sCD14 could be prognostic in ALS and elevated compared with FTD, Alzheimer’s disease or immune-mediated neuropathy.^68^ Urinary dilution was not considered when quantifying sCD14 in urine. In another study, sCD14 in the CSF of patients at baseline was reduced, and lower levels were prognostic.^77^ Larger studies are needed to determine the usefulness of urinary sCD14 as an ALS biomarker.

## Urinary metabolome

### Considerations regarding metabolite candidates as ALS biomarkers

Metabolites are small molecules of less than 1.5 kDa^201^ that are end products of cellular or organ processes. Since the kidneys do an extraordinary job of concentrating certain metabolites from the blood, some compounds that are far below the limit of detection in the blood are well above the detection limit in urine.^201^ Although there are variations in concentration of these components, due to diet, sex and time of day,^202^ relative to other biofluids such as CSF^203^ or saliva,^204^ urine contains significantly more compounds (5–10×) and exhibits significantly more chemical diversity (2–3×). Table 2 shows current metabolomic biomarkers for ALS including urinary markers.

**Table 2:** Metabolite (less than 1.5 kDa) biomarkers of ALS in biofluids

Name of sub-pathway or metabolite	Pathological association	Biofluid	Change in ALS	Detection technique	Link to disease
Glutamate metabolism	Neurodegeneration	Plasma	Increase	LC-MS	Plasma amino acids Linked to early ALS.^205^
Nucleotide metabolites	Neurodegeneration	Serum	Increase Decrease	LC-MS	Higher in ALS than healthy controls.^206^
Energy metabolites: glycolysis, gluconeogenesis	Energy metabolism	Serum/CSF	Increase	Colorimetric/LC-MS	Higher in ALS than healthy controls in CSF^207^ and serum.^206^
Creatine metabolites	Muscle degeneration	Serum	Increase	LC-MS	Higher in ALS than healthy controls.^206^
Creatinine	Muscle degeneration	Plasma	Increase	Colorimetric assay	Prognostic/not prognostic.^208-212^
Diacylglycerols	Lipid metabolism	Serum	Increase	LC-MS	Higher in ALS than healthy controls.^206,213^
Phosphatidylcholines: phosphatidylethanolamine	Lipid metabolism	Serum	Decrease	LC-MS	Related to progression^213^ and maybe diagnostic.^214^
Ceramide	Lipid metabolism	Serum	Decrease	LC-MS	Higher in ALS than healthy controls.^206,213^
8-Hydroxydeoxyguanosine	Oxidative stress	Serum, CSF, urine	Increase	ELISA HPLC/colorimetric electrodes	Serum: no clear association.^163^ Urine: correlated to ALSFRS-R.^215^ not correlated to ALSFRS-R.^216^
Uric acid	Oxidative stress	Serum	Increase	Enzymatic-colorimetric method	High Uric acid linked to slow ALS progression.^217^
Total antioxidant status	Oxidative stress	Serum	Increase	Colorimetric assay	Higher in ALS than controls.^218^
Glutathione metabolites	Oxidative stress	Whole blood	Increase	Colorimetric assay	Higher in ALS than controls.^218^
4-Hydroxynonenal	Oxidative stress	CSF, serum	Increase	ELISA, colorimetric assay	Correlated to disease^219^ and ALSFRS-R.^163^
Neopterin	Immune system	Urine	Increase	HPLC-UV, ELISA,	Prognostic.^220^ Not prognostic.^40^ Correlated to ALSFRS-R and increases longitudinally over disease progression.^40^

HPLC, high-performance liquid chromatography; LC, liquid chromatography; MS, mass spectrometry; UV, ultraviolet.

### Metabolites related to ALS pathology

Metabolites dysregulated in CSF and or plasma/serum and linked to ALS pathology have been described, but these are rarely investigated in urine (Table 2). Those in CSF and serum/plasma include altered amino acid metabolism, e.g. excitatory amino acid glutamate,^205,218^ and those associated with muscle loss (e.g. creatinine kinase) altered carbohydrate metabolism (e.g. glycan metabolites and energy metabolites such as glucose), short-chain fatty acids (e.g. acyl carnitine),^206,207^ co-factors and vitamin and nucleotide metabolites.^221^ Other groups of metabolites altered in ALS include those related to antioxidant defence such as glutathione metabolites^206^ and those altered in lipid metabolism.^213^

A major issue with most of the studies is a discordance in collection protocols and how each is related to the clinical stage of disease, including time from diagnosis, and relationship to ALSFRS-R.^222^ Meta-analysis including CSF and serum showed 16 pathways altered in ALS,^221^ including caffeine metabolism, aminoacyl-tRNA biosynthesis and valine, leucine and isoleucine biosynthesis. However, it is not clear in the metabolomics studies if the samples were all taken from ALS patients with similar ALSFRS-R and time from diagnosis. Notable exceptions are single metabolite studies focused on oxidative stress such as urate and 8-hydroxydeoxyguanosine (8-OHdG), where correlation to ALSFRS-R and relationship to prognosis and progression are reported.^215,217^

Creatinine (113.12 Da) is a breakdown product of creatine phosphate and reflects the creatine pool and the impaired uptake of creatine into muscle cells.^223^ Creatinine is released from muscle at a constant rate, resulting in a stable plasma concentration and is freely filtered at the glomerulus and secreted by the proximal tubules.^223,224^ Creatinine clearance is commonly determined from a 24-h collection of urine.^223^ Alternative excretory pathways through the gut may occur^224^ especially when the kidney is not working. Total creatinine excretion in the steady state is dependent on muscle mass, and day-to-day creatinine excretion remains constant for an individual and is related to lean body weight, age, sex and ethnicity. In general, men excrete 20–25 mg creatinine/kg body weight/day, whereas women excrete 15–20 mg/kg/day and may be affected by diet, for example, eating a large meal of cooked meat, which may also result in a substantial increase in serum creatinine.^224^

Studies have also indicated that plasma creatinine levels may be a simple biomarker for ALS muscle wasting and decreased serum creatinine at baseline is prognostic for poor survival and associated with an aggravation of the disease progression.^208-211^ A meta-analysis of 14 studies of plasma creatinine concluded that mortality was higher if creatinine was lower than the median, but, noted caution, because confounding factors such as age, sex and body mass index were not uniformly included in some analyses and are known to be associated with ALS and plasma creatinine levels.^225^ Another study that included all the covariates did not find any relationship between plasma creatinine and survival nor progression rate but did find a correlation with the ALSFRS-R at baseline.^212^ Interestingly, Mitsumoto *et al*.^210^ expected there to be higher levels of urinary creatinine as muscle waste, but to date, this has not been found.^39^ It is possible the reduction in serum creatinine is reflective of muscle mass rather than muscle breakdown, but the variability and the known covariates make it difficult to determine reliability as a progression and prognostic biomarker.

A recent study focusing on lipids showed that triglycerides were increased and ceramides were decreased in ALS compared with controls, and phosphatidylethanolamine decreased over disease progression but there was again no reported relationship to ALSFRS-R.^213^ Covariates, such as BMI, sex, time of sample collection and presence of diabetes, are not uniformly used in every analysis, which could account for differences in top significant metabolites across reported literature.^206,218,221^ There is one study that looked at blood taken 5 years before diagnosis^214^ from over 200 000 people including 260 individuals who developed ALS, which found no significant metabolite associated with ALS diagnosis, once all the covariates were included in the analysis, and taking into account multiple comparisons. However, there was a trend in decreasing metabolites such as nucleosides, triacylglycerols, urate and phosphatidylcholines^214^ associated with developing ALS. Interestingly, there was no correlation to metabolites previously detected in ALS patients such as in the meta-analysis of Blasco *et al*.^221^ Metabolites dysregulated in the urine of ALS patients include 8-OHdG^215^ and neopterin.^40,220^

### Metabolites related to oxidative stress in ALS

Several metabolites have been suggested as biomarkers of oxidative stress for ALS. An oxidized nucleoside of DNA, 8-OHdG (283.3 Da), is a widely studied oxidized metabolite of DNA damage present in urine, as well as CSF and blood. In an ALS study, urinary 8-OHdG (corrected by creatinine) was increased in compared with healthy controls, was correlated to the ALSFRS-R and was increased at a measurable rate over a 1-year study.^215^ Although higher in ALS than in healthy individuals, another study found no association of urinary 8-OHdG with the ALSFRS-R nor change over progression.^216^ Other research has found higher levels in the plasma of ALS patients compared with healthy controls and no difference between slow and fast progressors over time.^163^ Interestingly, a study of healthy individuals found that there was a correlation between urinary 8-OHdG and older age and gender.^226^ Urinary 8-OHdG has been used in a pre-clinical study as a pharmacodynamic biomarker^227^ but not in clinical trials. Further work needs to be done on a larger sample population to determine if urinary 8-OHdG is prognostic for survival in ALS and could be used as a predictive biomarker for treatments targeting oxidative stress.

High uric acid (168.11 Da) in serum, which is a final product of purine metabolism and an oxidative stress marker, has been linked to slower disease progression in ALS,^217^ and low levels of uric acid at diagnosis are prognostic in ALS, especially for males. A Phase 2 randomized, double-blind placebo-controlled trial using inosine to increase urate successfully increased serum urate, but no improvement was seen in the ALSFRS-R; however, this was a small safety and tolerability trial.^228^ 4-Hydroxynonenal (156.22 Da), an oxidative stress marker produced by lipid peroxidation, also showed promise,^163,219^ with higher baseline levels correlated to worse symptoms 18 months later, faster progressing patients having higher levels of 4-hydroxynonenal. Urinary uric acid and 4-hydroxynonenal have not yet been reported in ALS patients.

### Metabolites related to immune dysfunction in ALS

A downstream product of cytokine signalling, urinary neopterin, is a small metabolite of 253.21 Da and a promising pro-inflammation marker in ALS.^40^ Neopterin derives from guanosine triphosphate and is part of the pteridine family,^229,230^ which are pyrazino-pyrimidine compounds whose biological activity is dependent on chain substituents as well as the oxidation state of the ring. Tetrahydrobiopterin, a reduced pteridine that exhibits biological activity, functions as a co-factor of inducible nitric oxide synthase production^230^ and regulates apoptotic death by nitric oxide synthesis.^231^ However, tetrahydrobiopterin is unstable and easily oxidized to dihydrobiopterin and then biopterin.^230,232^ Neopterin is the oxidized product of 7,8-dihydroneopterin, and both biopterin and neopterin are stable in urine.^230,232^ Other pterdines include xanthopterin, isoxanthopterin, 6,7-dimethylpterin, 6-biopterin, 6-xydroxymethylpterin, pterin and pterin-6-carboxylic acid, which have not been extensively investigated as biomarkers in urine and not implicated in ALS.

In 1967, Sakurai and Goto^233^ isolated 25 mg of neopterin from 500 L of human urine. Neopterin is fluorescent in urine and detectable by HPLC at 353 nm excitation and 438 nm emission wavelengths.^234^ Neopterin [2-amino-4-oxo-6-(d-erythro-1,2,3,trihydroxypropyl)-pteridine] and its reduced form, 7,8-dihydroneopterin, are produced in large amounts by activated monocytes, macrophages and dendritic cells,^235,236^ after stimulation with IFN-γ and, to a lesser extent, TNF-α.^237^ Since microglia are the resident macrophages of the CNS, it has been assumed that they produce neopterin, as they respond to IFN-γ,^238^ and neopterin has been detected in the CSF.^239,240^ There is some *in vitro* evidence that microglia and neurons release neopterin. The concentration of neopterin reflects the presence of IFN-γ in body fluids, which makes it a sensitive marker of cell-mediated immunity.^241^

Evidence from animal models^242,243^ suggests that in response to ALS pathology, including protein aggregation, there is an anti-inflammatory response, which then shifts to pro-inflammatory as the anti-inflammatory process is overwhelmed with coping with accumulating protein aggregation. Microglia become pro-inflammatory as part of the activated microglial response^244^ and induce the release of neurotoxic factors from astrocytes that can kill motor neurons.^245,246^ In non-neuronal cells outside of the CNS, the pro-inflammatory state is evidenced by a switch to T-helper types 1 and 17 cells, as well as induction of cytotoxic CD8 cells, inflammatory monocytes and natural killer cells.^247^ This cascade also results in T-helper type 1 cell release of pro-inflammatory cytokines such as ILs and IFN-γ.^248^

Neopterin was first detected in some serum and CSF samples from ALS patients in 1993 and in 2020 in urine via ultraviolet HPLC,^220^ where it was suggested to be a prognostic marker. In a more recent analysis, undertaken by the authors, using an ELISA, and a smaller number of samples, neopterin was not prognostic, although correlated to the ALSFRS-R.^40^ Interestingly, neopterin increased at a measurable rate over disease progression,^40^ and it was suggested to be a candidate predictive marker of pro-inflammation in ALS.^40^ Further large studies to determine if neopterin is a valid predictive biomarker useful in clinical trials that influence the inflammatory state in ALS are required.

## Reflections and limitations

**Figure 2:**
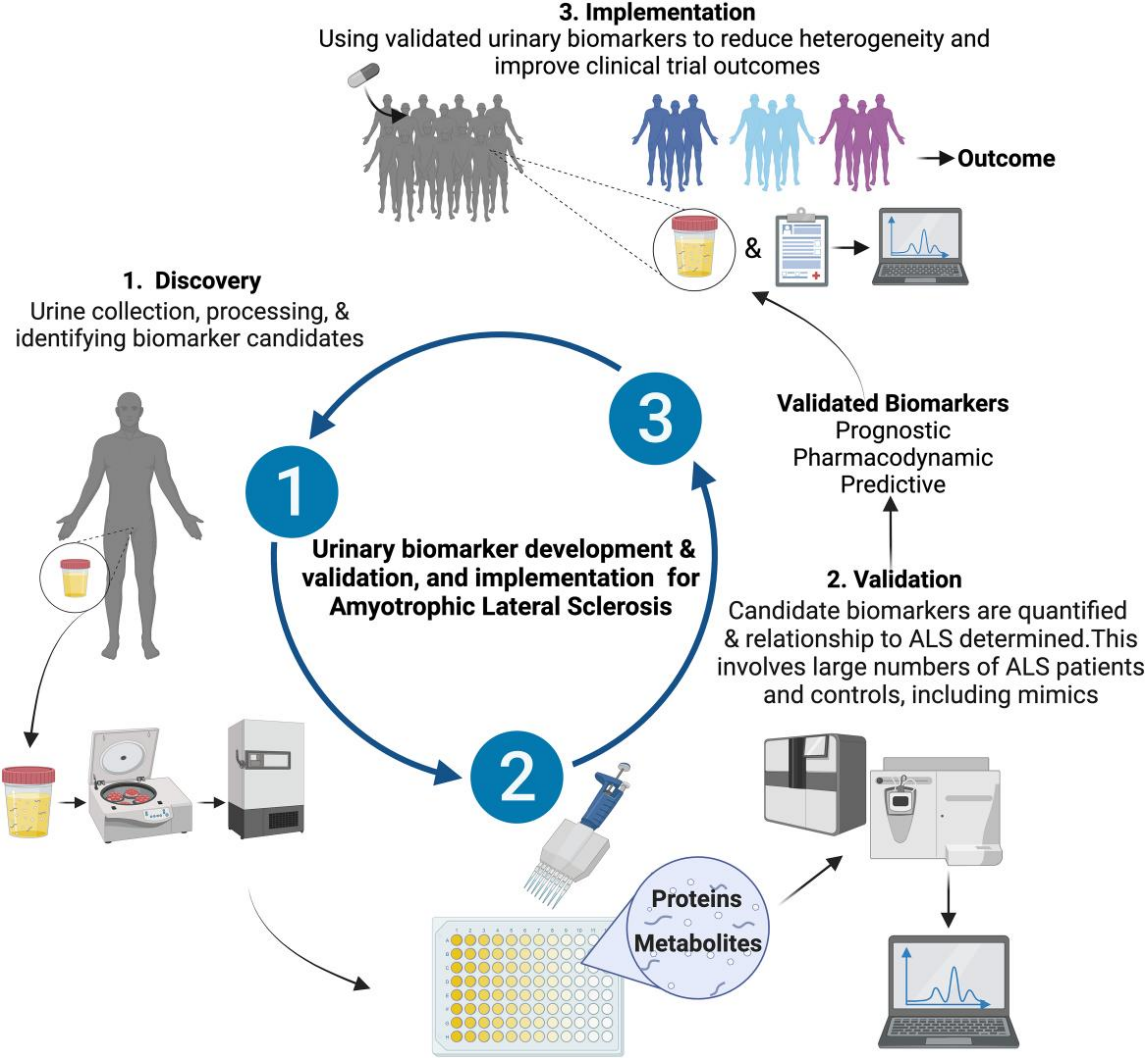
**Urinary biomarker development and implementation for ALS**. The urinary biomarker development includes discovery, validation and utilization. Urine samples are collected from people with ALS and controls for discovery-type experiments. Biomarkers are isolated, measured and compared with clinical characteristics and pathology to determine if a possible candidate biomarker. The biomarkers are then validated across larger cohorts of ALS and controls, including mimic diseases. Validated prognostic, pharmacodynamic and predictive biomarkers can then be utilized to reduce heterogeneity and determine efficacy in clinical trials and determine outcomes (drug was a success or not).

The relationship between pathological features in ALS and what is happening to motor neurons and support cells should be reflected in a candidate biomarker. Since the CSF is close to the site of injury, it has been expected to be the most useful biomarker, although other biomarkers are deemed valid if correlated to that found in CSF.^91^ Urinary biomarkers that reflect pathological processes in ALS should be examined, as obtaining urine is less invasive for ALS patients than CSF. Multiple components of urine are ripe for investigation as biomarkers for MND (Fig. 2) that can improve the chance of finding treatments. However, as a biomarker source, urine is not without limitations.

### Heterogeneity

Urinary protein and metabolite biomarkers can be affected by the time of collection, hydration status, urinary pH, kidney function, sex, age, diet and other disease states.^32-34^ Degradation and proteolysis can occur while the urine is stored in the bladder and in urine samples upon storage.^32-34^ For example, in a quantitative report on the urinary proteome, inter-individual variability exceeded 47% and intra-individual variability exceeded 45%.^32^ Metabolites in normal urine can vary even more than the proteome^202^; for example, using the large data from the National Health and Nutrition Examination Survey (2698 individuals), urinary phytoestrogen concentration was highly variable across the population, even where age, sex, lifestyle, race and poverty were used to reduce variability.^249^

The type of urine collection could be another source of variability. Although 24-h urine samples are the gold standard for biomarker measurement, it is often not practical, spot urine samples, when corrected for urinary dilution is seen as an adequate surrogate.^250^ Hydration is a source of urine variability in spot urine samples, and the use of creatinine as a hydration correction factor^251^ has been in clinical medicine for over 40 years.^252^ This is because creatinine has a steady state of excretion, but creatinine is effected by muscle mass, diabetes and meat (protein) intake.^224^ Nevertheless, the World Health Organization has defined cut-off values for very dilute (less than 0.3 mg/ml) and very concentrated (higher than 3.0 mg/ml) in urine^252,253^ In our 2017 and 2022 studies,^39,40^ applying these limits for spot urine creatinine for urinary dilution protection to biomarkers did not preclude detection of prognostic value or association with ALSFRS-R and progression. In addition, there was no diurnal variation of the candidate biomarkers. However, osmolality, which is not related to muscle mass nor diabetes status, should be considered as a correction factor, and in a large population study, osmolality was shown to be acceptable.^254^

### Recommendations for standardizing spot urine collection, processing and use in biomarker analysis

Standardized protocols are essential^255^ to reduce the variability of the urinary proteome and metabolome in spot samples. Urine from those with renal or bladder abnormalitie**s** or uncontrolled diabetes should be screened out of the study. Our experience^38-40^ and the literature^36,255,256^ suggest those interested in urinary biomarkers for ALS use the following procedures:

- Urine collection should be mid-stream.
- The time between collection and sample processing is no more than 4 h, with samples stored on ice.
- Urinalysis or urine dipsticks should be used to test for blood, high glucose, high bilirubin, high pH and the presence of high leukocytes.^255^ Abnormalities should be reported to the physician immediately and preclude the urine being used.
- Although stabilizers such as boric acid and sodium azide can be added, literature^36,256^ and our experience^38-40^ suggest that centrifugation at 2000*g* at 4°C is preferable to remove cellular debris that may interfere with assays. If undertaking mass spectrometry analysis, a list of contaminant marker proteins can be used to screen out samples.^256^
- After centrifugation, the urinary supernatant should be aliquoted into storage vials and stored at −70°C to reduce the necessity of going through freeze–thaw cycles.
- Each biomarker once identified should be tested for stability at room temperature and at 4°C over at least 72 h. Diurnal stability and effect of ultraviolet light should also be undertaken with the goal of having as less variability as possible.
- Once standardized protocols are in place, each biomarker should be checked for association with age and sex and, in the case of metabolome, diet.^202^
- Consider using osmolality to correct for urinary dilution.

## Conclusions

Standardizing urine collection and protocols for testing biomarkers will increase the usefulness of urine as potential prognostic, progression (monitoring) and predictive biomarkers for ALS. As listed above, there are many possible biomarkers related to ALS pathology, found in other biofluids, that can be investigated in urine. At present, the most promising urinary biomarker candidates include p75^ECD^, neopterin, titin and 8-OHdG. Each candidate should be validated in large cohorts that include healthy controls and disease mimics and candidates validated across laboratories. The recent inclusion of urine from ALS patients in large biobanks is encouraging. For example, the National Institute of Health funded Clinical Research in ALS and Related Disorders for Therapeutic Development biobank (https://create.rarediseasesnetwork.org/resources/researchers-clinicians/create-biorepository). After validation, the biomarkers can then be classified as prognostic, pharmacodynamic or predictive (or a mixture). For clinical trials, urinary biomarkers and panels of biomarkers for ALS can then be utilized to reduce heterogeneity and to determine if a potential ALS treatment is useful or not (Fig. 2). Ideal candidates should also be able to describe the pathological processes and to be used to tailor treatments (when available) for individuals with ALS. An example is a pro-inflammatory biomarker that could be used to detect those that may respond to an anti-inflammatory treatment.

## Data Availability

Data sharing is not applicable to this article as no new data were created or analysed in this study.
