# Correction to: Urinary biomarkers for amyotrophic lateral sclerosis: candidates, opportunities and considerations

**DOI:** 10.1093/braincomms/fcad334

**Published:** 2023-12-11

This is a correction to: Mary-Louise Rogers, David W Schultz, Vassilios Karnaros, Stephanie R Shepheard, Urinary biomarkers for amyotrophic lateral sclerosis: candidates, opportunities and considerations, Brain Communications, Volume 5, Issue 6, 2023, fcad287, https://doi.org/10.1093/braincomms/fcad287

The originally published version of this manuscript has been corrected.

Section heading “Progranulin, vascular endothelial growth factor, TBF-beta and ferritin/transferrin” has been corrected to read “Progranulin, vascular endothelial growth factor, TGF-beta and ferritin/transferrin”.

In addition, there was an error in the following sentence, whereby “−7°C” should have been given as “−70°C”:

“After centrifugation, the urinary supernatant should be aliquoted into storage vials and stored at −7°C to reduce the necessity of going through freeze–thaw cycles.”

The sentence has been corrected to read as follows:

“After centrifugation, the urinary supernatant should be aliquoted into storage vials and stored at −70°C to reduce the necessity of going through freeze–thaw cycles.”

